# Investigation of Position-Dependent Signal Propagation Delay in Large-Pitch AC-LGAD Using a Two-Dimensional Transmission-Line Model

**DOI:** 10.3390/s26175566

**Published:** 2026-09-02

**Authors:** Houqian Ding, Weiyi Sun, Xiang Li, Mengzhao Li, Zhijun Liang, Mei Zhao, Tianyuan Zhang, Yuan Feng, Xinhui Huang, Yunyun Fan, Tianya Wu, Xuan Yang, Bo Liu, Wei Wang, Gaobo Xu, Ming Qi

**Affiliations:** 1 Department of Physics, Nanjing University, Nanjing 210093, China; houqianding@smail.nju.edu.cn (H.D.);; 2 Institute of High Energy Physics, Chinese Academy of Sciences, 19B Yuquan Road, Beijing 100049, China; 3 University of Chinese Academy of Sciences, 19A Yuquan Road, Beijing 100049, China; 4 School of Integrated Circuits, Dalian University of Technology, Development Zone Campus, Dalian 116023, China; xiangli@ihep.ac.cn; 5 School of Information Engineering, Nanchang University, Nanchang 330031, China; 6 Shanghai Institute of Applied Physics, Chinese Academy of Sciences, Shanghai 201800, China; 7 School of Physics, Nankai University, Tianjin 300071, China; 8 Institute of Microelectronics, Chinese Academy of Sciences, 3 Beitucheng West Road, Beijing 100029, China

**Keywords:** AC-LGAD, LGAD, silicon detector, timing detector, transmission-line model

## Abstract

This paper presents a study of position-dependent signal propagation delay in large-pitch pixelated AC-coupled Low-Gain Avalanche Detectors (AC-LGADs). In AC-LGADs, a continuous resistive $N^{+}$ layer and segmented AC-coupled readout electrodes enable charge sharing and simultaneous timing and position measurements. However, lateral signal transport in the resistive layer can introduce a position-dependent delay in the measured signal arrival time. In this work, an IHEP-designed pixel AC-LGAD was characterized using a two-dimensional picosecond laser scan. The measured leading-edge arrival time shows an approximately linear dependence on an effective propagation distance, with a delay slope of about 194.7±1.3ps/mm for the tested device. After applying a position-dependent delay correction, the sigma of the combined arrival-time distribution over the scanned region is reduced from 88.3 ps to 48.6 ps. To interpret the observed delay, an equivalent two-dimensional lossy transmission-line model is developed for the continuous resistive layer. The model provides a semi-quantitative description of the leading-edge delay and indicates that, within the measured signal bandwidth, the transport is dominated by the resistive term and is therefore dispersive and diffusion-like. A distributed SPICE network including the pad-area response and capacitive charge sharing provides a complementary circuit-level cross-check of the approximately linear distance dependence. These results quantify the propagation-induced timing delay in large-pitch AC-LGADs and provide guidance for timing correction and future optimization of the resistive-layer sheet resistance.

## 1. Introduction

Low-gain avalanche detectors (LGADs) have become one of the most important silicon sensor technologies for precision timing. Their excellent timing performance makes them attractive for high-luminosity collider experiments and for other applications requiring fast signal formation [1,2]. AC-coupled LGADs (AC-LGADs) extend this concept by introducing a continuous gain layer and a continuous resistive $N^{+}$ layer beneath segmented AC readout pads [3,4,5]. This design enables charge sharing among multiple pads and therefore allows simultaneous timing and position measurements with a high fill factor [5,6].

A position-dependent delay is an intrinsic concern in AC-LGADs because the signal induced on the resistive $N^{+}$ layer propagates laterally before being capacitively coupled to the readout electrodes [7,8]. Such an effect has been reported in FBK RSD sensors, where delays of the order of 300ps/mm were observed depending on the sheet resistivity and capacitive coupling [9]. A larger apparent delay, about 700ps/mm, was also reported in centimeter-scale BNL strip AC-LGAD sensors tested with a 120GeV proton beam, particularly in the non-metalized regions between strips [10]. Although position-dependent signal delays have been observed and corrected in both FBK and BNL AC-LGAD devices, a quantitative description that connects the observed delay to the distributed electrical parameters of large-pitch pixel AC-LGADs is still needed for timing correction and sensor optimization.

The main contribution of this work is to provide a quantitative study of the position-dependent leading-edge delay in a large-pitch pixel AC-LGAD and to relate this delay to the distributed electrical parameters of the sensor. Compared with previous studies on FBK RSD sensors and BNL strip AC-LGADs, this work develops an effective two-dimensional lossy transmission-line model to describe the lateral signal transport in the resistive $N^{+}$ layer. This model connects the observed delay slope to the sheet resistance, coupling capacitance, and sheet inductance, and is used to evaluate how the resistive-layer sheet resistance affects the trade-off between charge sharing, signal attenuation, and timing uniformity. In addition, a distributed SPICE network including the four readout pads and capacitive charge sharing is introduced as a complementary circuit-level cross-check. These results provide a practical basis for position-dependent timing correction and for future optimization of large-pitch AC-LGAD sensor designs.

## 2. AC-LGAD Sensor

**Figure 1:**
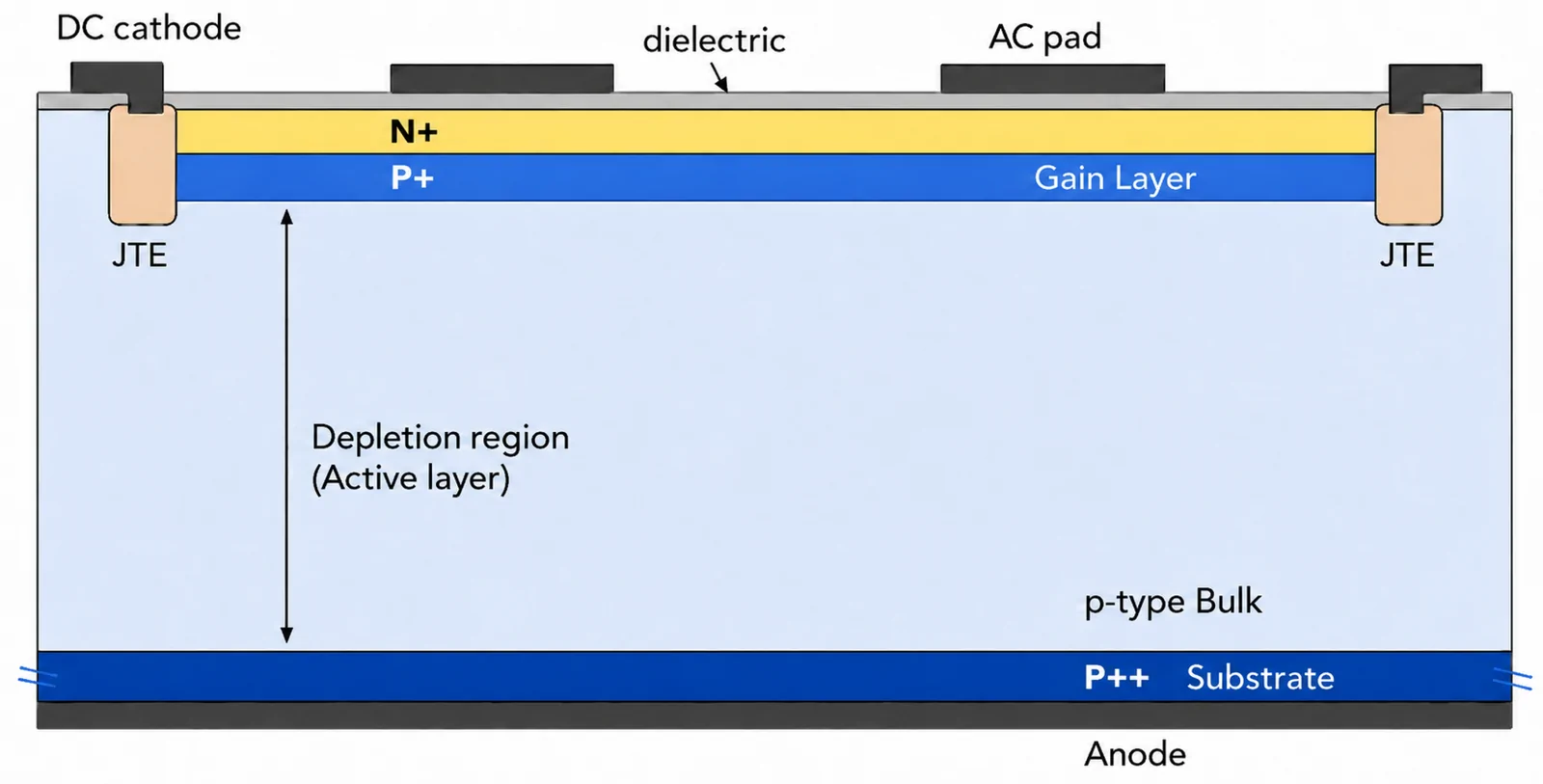
Schematic cross section of the AC-LGAD used in this work. The continuous gain layer and resistive layer are combined with segmented AC readout pads.

The AC-LGAD sensor used in this study was designed at the Institute of High Energy Physics (IHEP, Beijing, China) and fabricated by the Institute of Microelectronics (IME, Beijing, China) [11,12]. In this work, the laser measurements and timing analysis were performed on the device. It should be noted that this device does not represent the optimized timing performance achievable with IHEP-developed AC-LGAD devices. A schematic cross section of the device is shown in Figure 1. The detector contains a continuous $P^{+}$ multiplication layer and a continuous resistive $N^{+}$ layer. The metal readout electrodes are segmented AC pads separated from the resistive layer by a thin dielectric layer, so the signal is read out capacitively.

**Figure 2:**
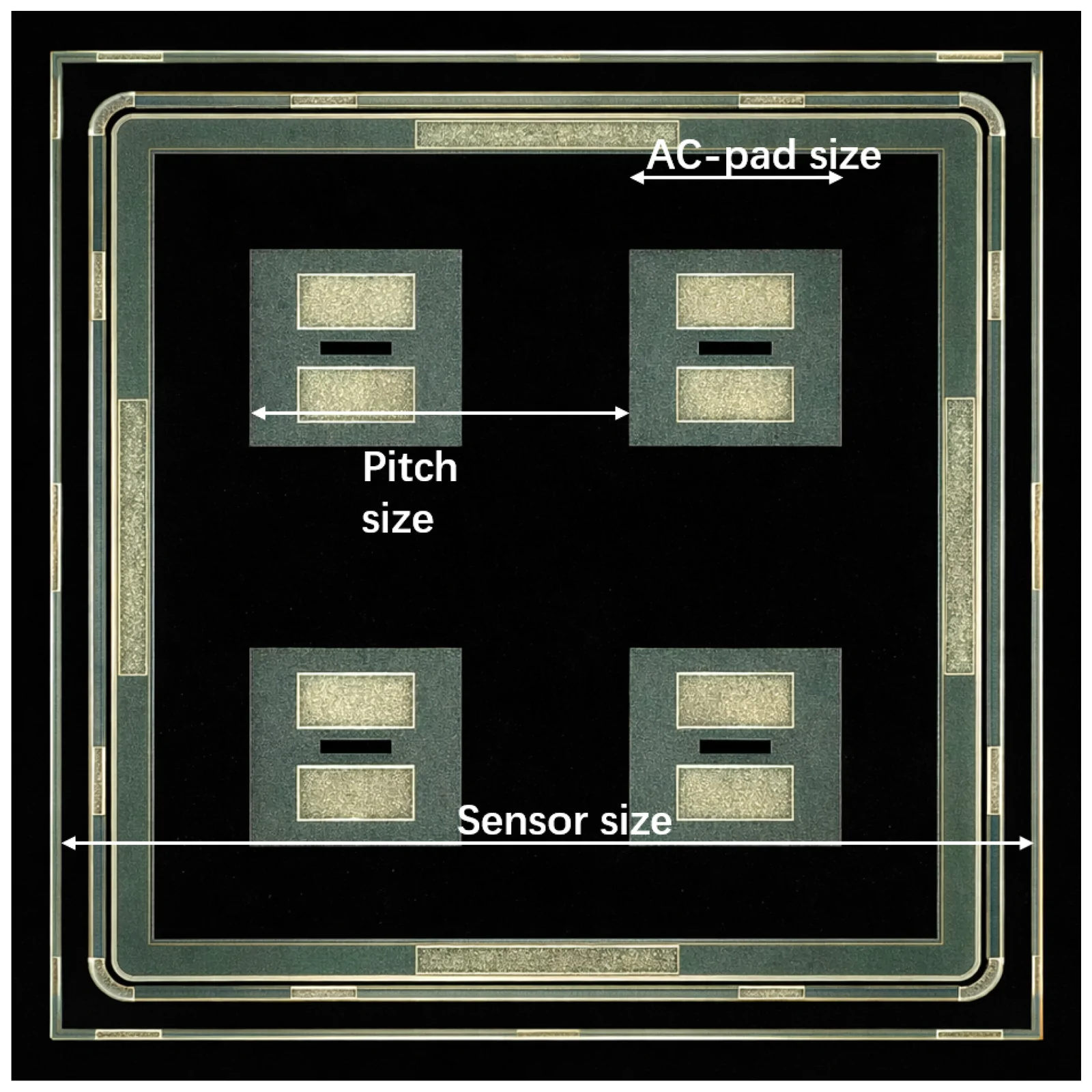
Photograph of the tested pixelated AC-LGAD. The outer rings are the DC ring and guard ring, and the inner region contains four AC readout pads.

A photograph of the tested device is shown in Figure 2. The sensor size is 6000μm×6000μm, the pitch size is 3000μm, and the AC pad size is 1500μm. The outer structures are the DC ring and the guard ring, while the inner region contains four pixelated AC pads. The active-layer thickness of the sensor is 50 μm.

## 3. Experimental Setup and Results

### 3.1. Picosecond Laser Test System

**Figure 3:**
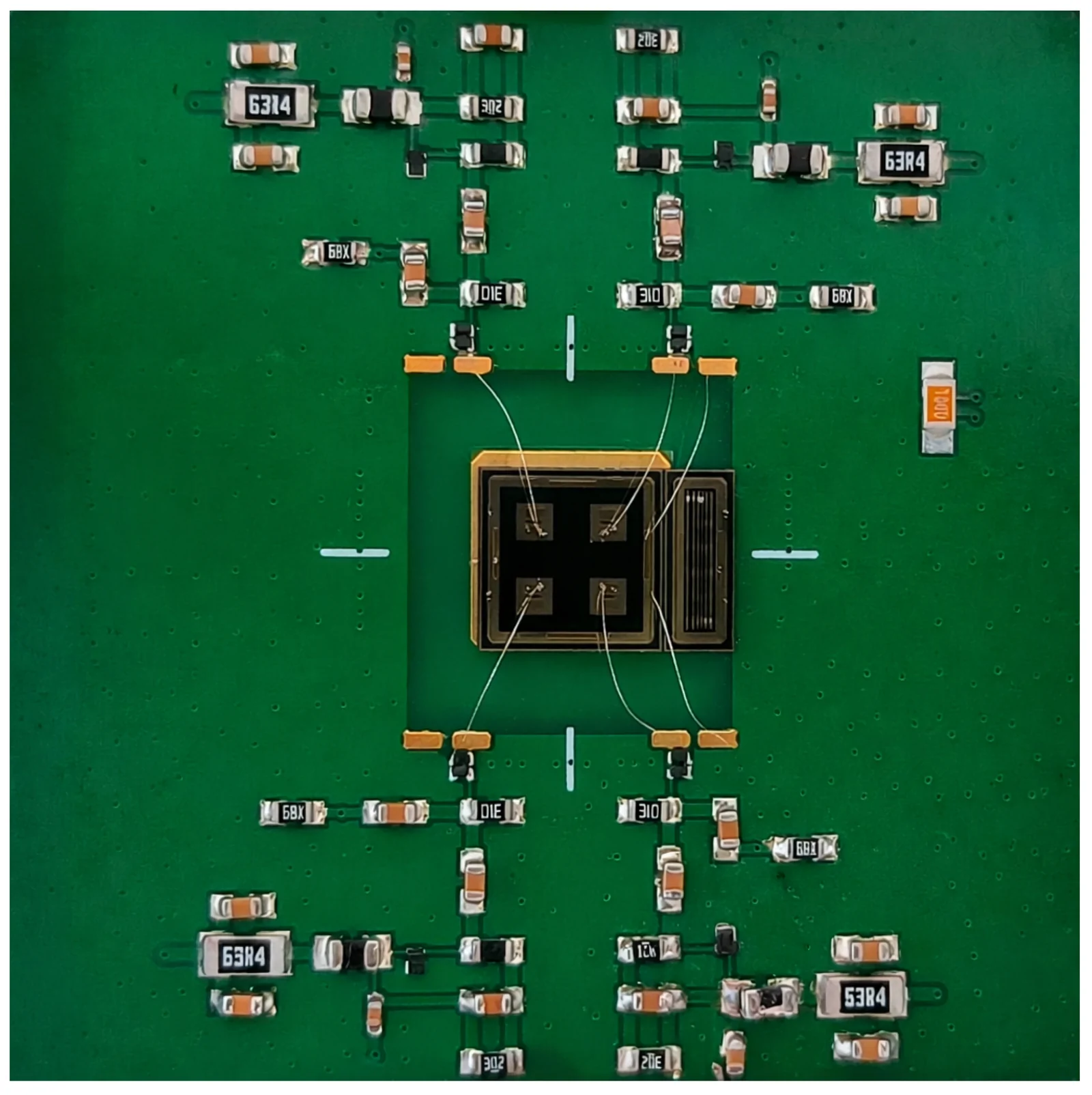
The four AC pads were wire-bonded to a four-channel readout board. The amplified signals were recorded by the oscilloscope.

The four-channel readout board was designed and manufactured according to the University of California Santa Cruz (UCSC) single-channel readout board [13]. The electrodes’ signals are recorded by the HDO9204 digital oscilloscope (Teledyne LeCroy, Chestnut Ridge, NY, USA) after the preamplifier and second-stage amplifier. The oscilloscope’s bandwidth is 2 GHz, and the sampling rate is 20 Gs/s for four channels. The AC-LGAD was mounted on a four-channel readout board, and the four AC pads were wire-bonded to the board, as shown in Figure 3.

**Figure 4:**
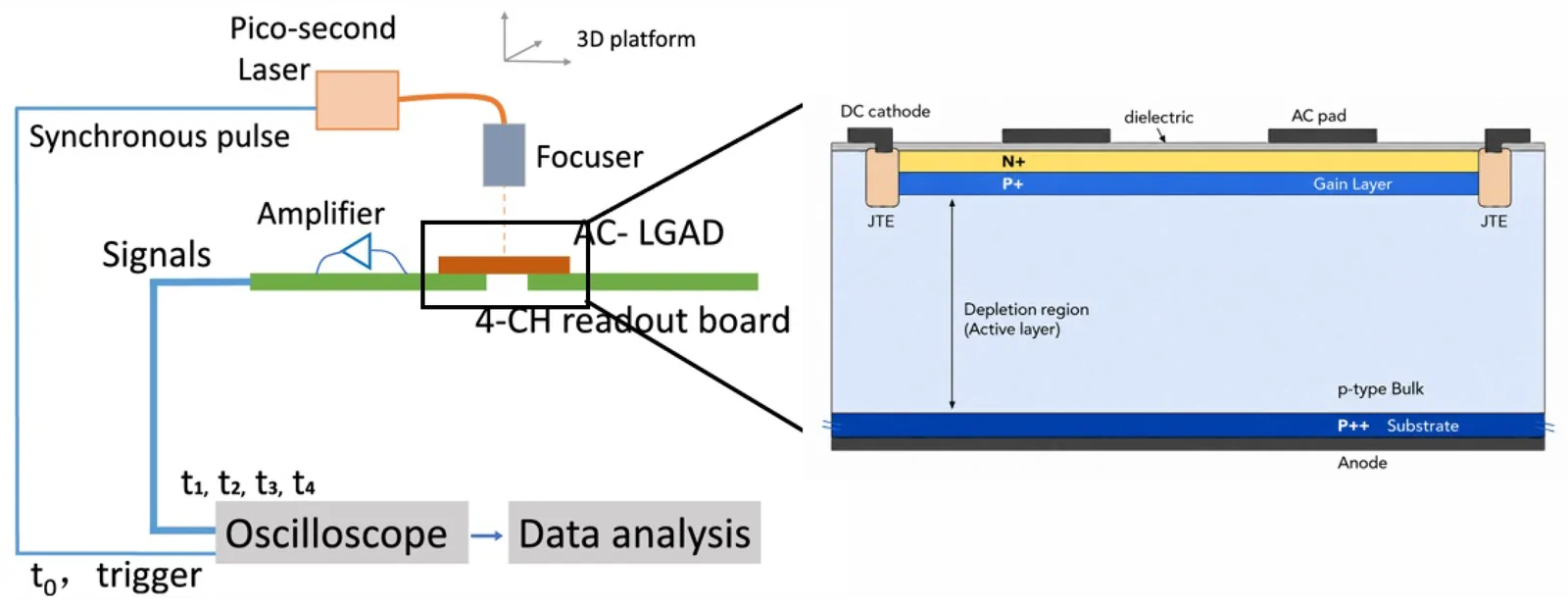
Schematic diagram of the picosecond laser TCT measurement setup.

Figure 4 shows the laser TCT experimental setup. A picosecond laser pulse with a wavelength of 1064 nm, generated by a laser system (Shanghai Precilasers Technology Co., Ltd., Shanghai, China), hit the Pixel AC-LGAD at a frequency of 20 MHz through a focuser. The laser intensity delivered to the sensor corresponded to an estimated charge deposition of approximately four minimum-ionizing particles (MIPs). The focuser is fixed on the positioning stage to achieve high-precision movement better than 1 µm. The laser spot size was smaller than 5 µm. The signals from the four pads were amplified by the on-board amplifiers and external pre-amplifiers, and then recorded by the oscilloscope through four channels. The synchronization output of the laser driver was connected to the external trigger input of the oscilloscope to provide a stable time reference. During the measurement, the DC ring and guard ring were grounded.

**Figure 5:**
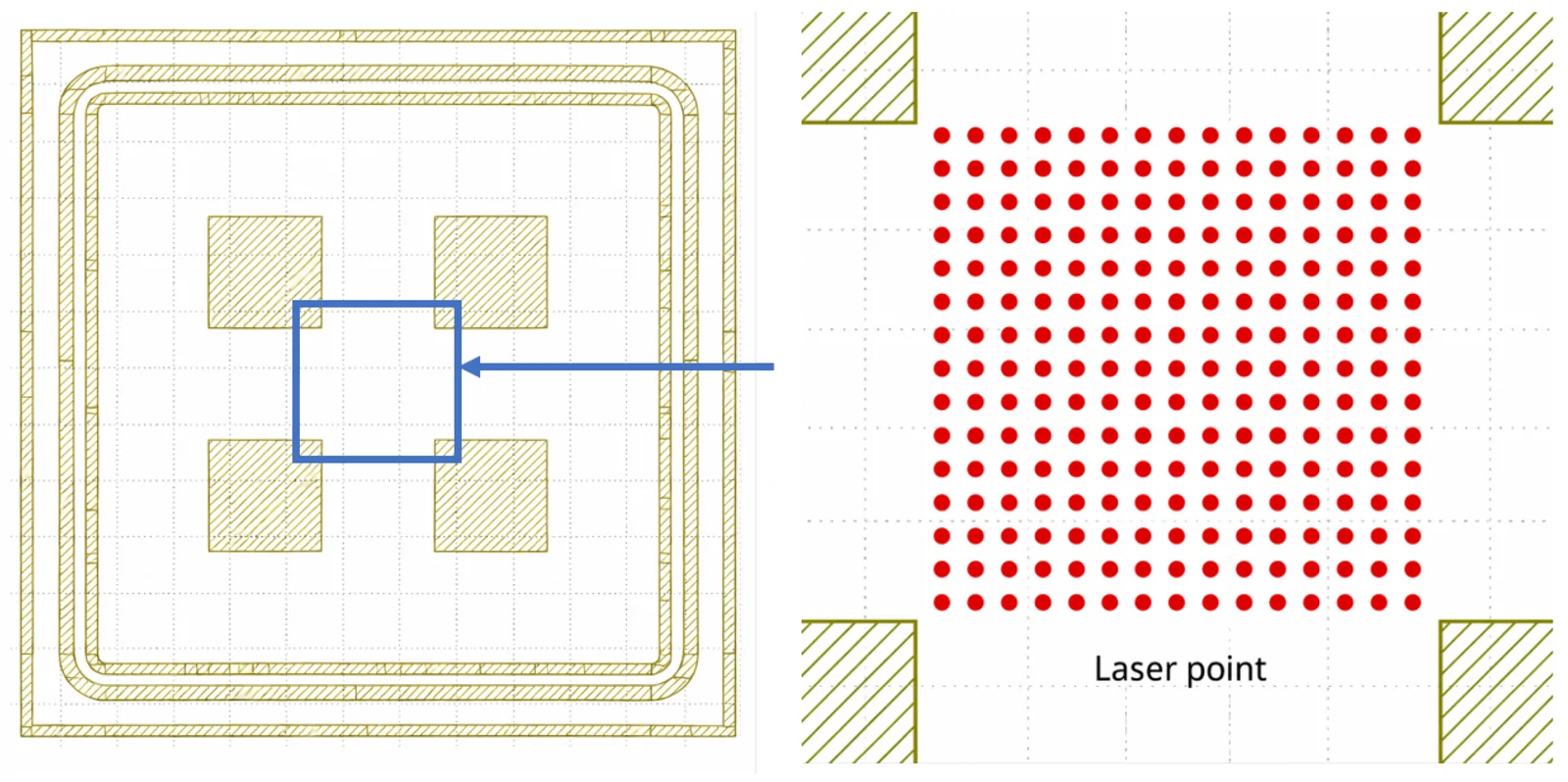
Schematic view of the 15×15 picosecond-laser scan used in this work.

With the sensor biased at −140 V and the laser focus optimized, a high-precision optical motion stage was used to scan the device over a 15×15 grid with a step size of 100 µm, as illustrated in Figure 5. Approximately 500 waveforms were recorded at each scan point. The laser spots are numbered from left to right and from bottom to top, ranging from X1Y1 to X15Y15.

### 3.2. Position-Dependent Waveforms and Arrival-Time Maps

**Figure 6:**
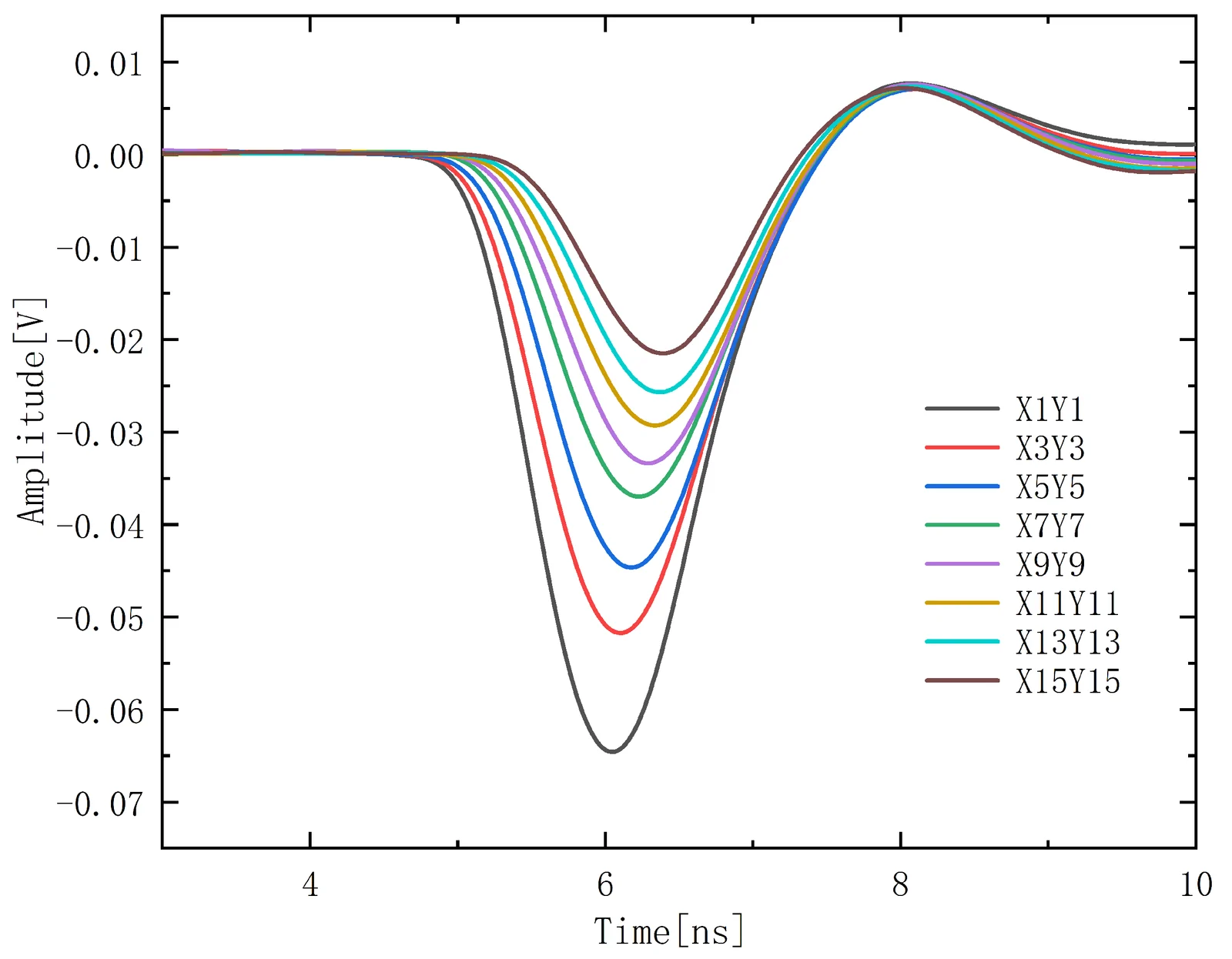
Measured waveforms for different laser positions along the diagonal direction, with the signal read out from the lower-left pad. Both the amplitude and the arrival time depend strongly on the hit position.

Figure 6 shows waveforms measured at different hit positions for the lower-left readout pad. Clear position dependence is observed. As the hit point moves away from the readout pad, the signal amplitude decreases, the leading edge becomes slower, and the pulse arrival time shifts to later values. This behavior is much stronger than the delay expected from a nearly lossless electromagnetic propagation picture, indicating that the lateral signal transport in the resistive layer plays the dominant role.

The signal arrival time is extracted using a constant fraction discriminator (CFD) algorithm at 40% level to the leading edge of the signal.

**Figure 7:**
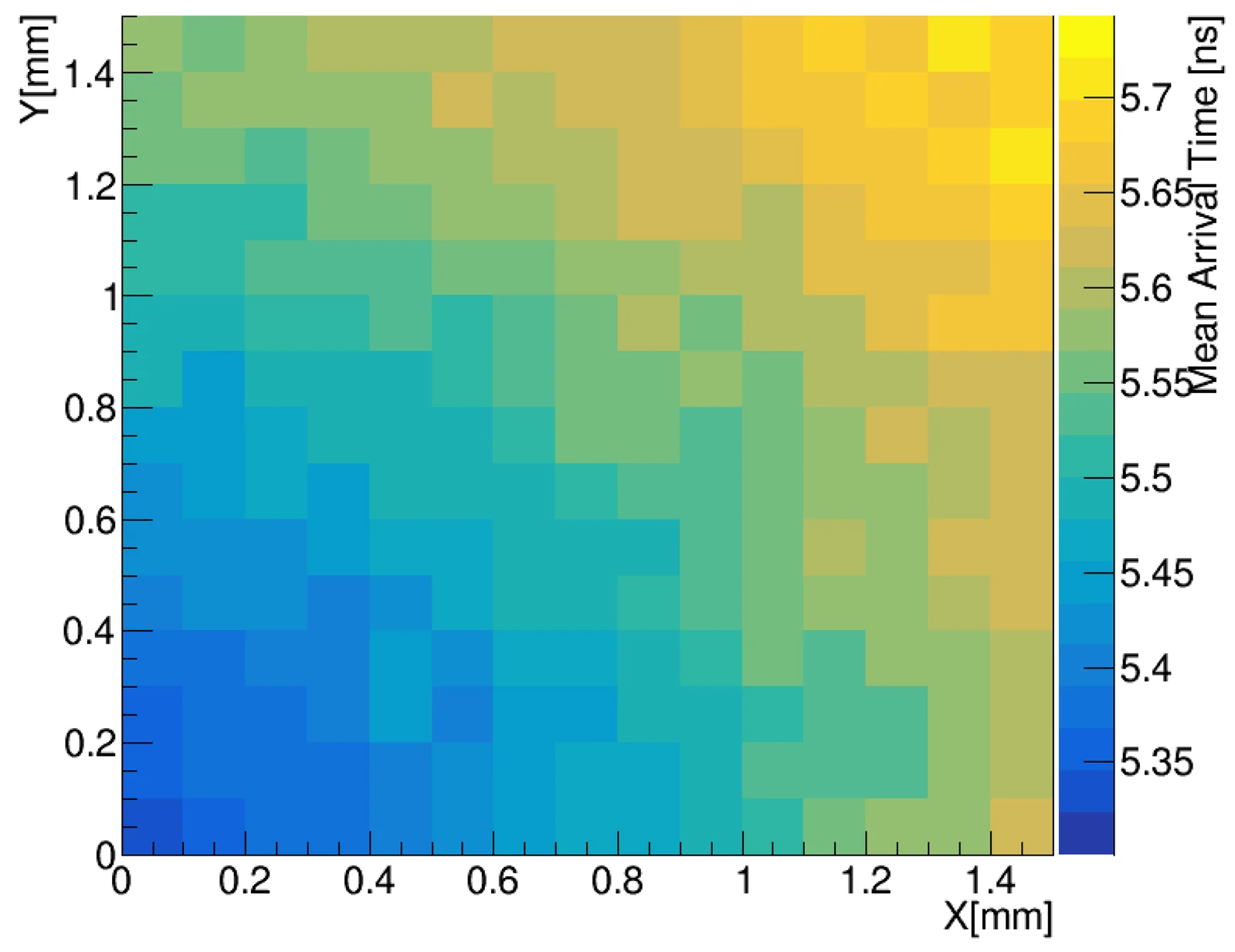
Two-dimensional map of the mean arrival time for the AC-LGAD.

The two-dimensional map of the mean arrival time for the signal read out by the lower-left AC pad is shown in Figure 7. In this case, the mean arrival time increases monotonically as the hit point moves away from the readout pad. For this sensor, the mean arrival time changes from about 5.34 ns at the nearest point to about 5.70 ns at the farthest point.

**Figure 8:**
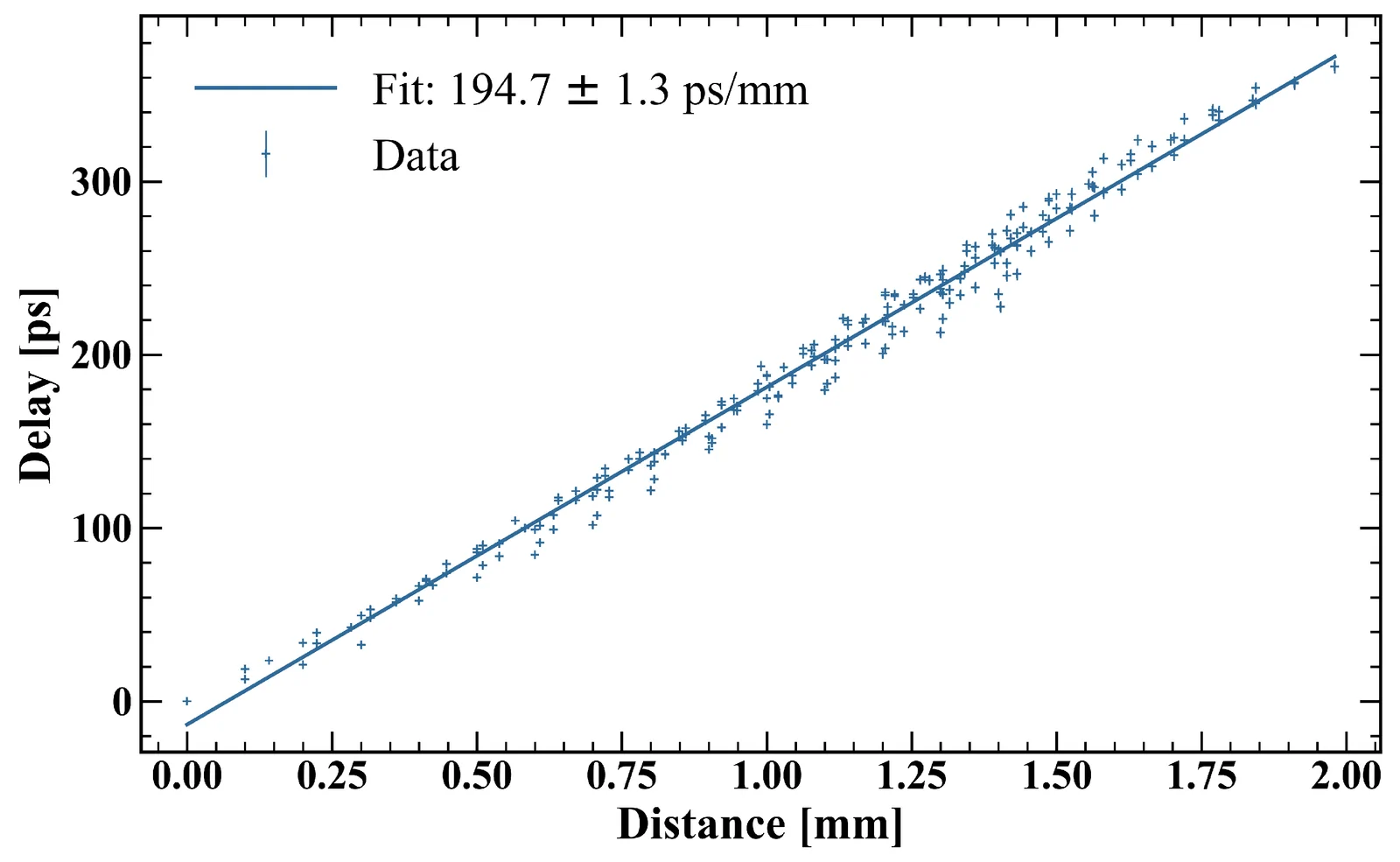
Relative arrival time delay of the device as a function of the effective one-dimensional propagation distance. The slope of the linear distribution is about 194.7±1.3ps/mm.

When the data are plotted as a function of the effective propagation distance, a clear linear trend is obtained, as shown in Figure 8. The effective propagation distance is defined as the shortest distance between the laser-spot center and the edge of the AC pad. The fitted delay slope is about 194.7±1.3ps/mm for the device. The strong linearity justifies the use of an effective one-dimensional distance to describe the dominant delay term.

**Figure 9:**
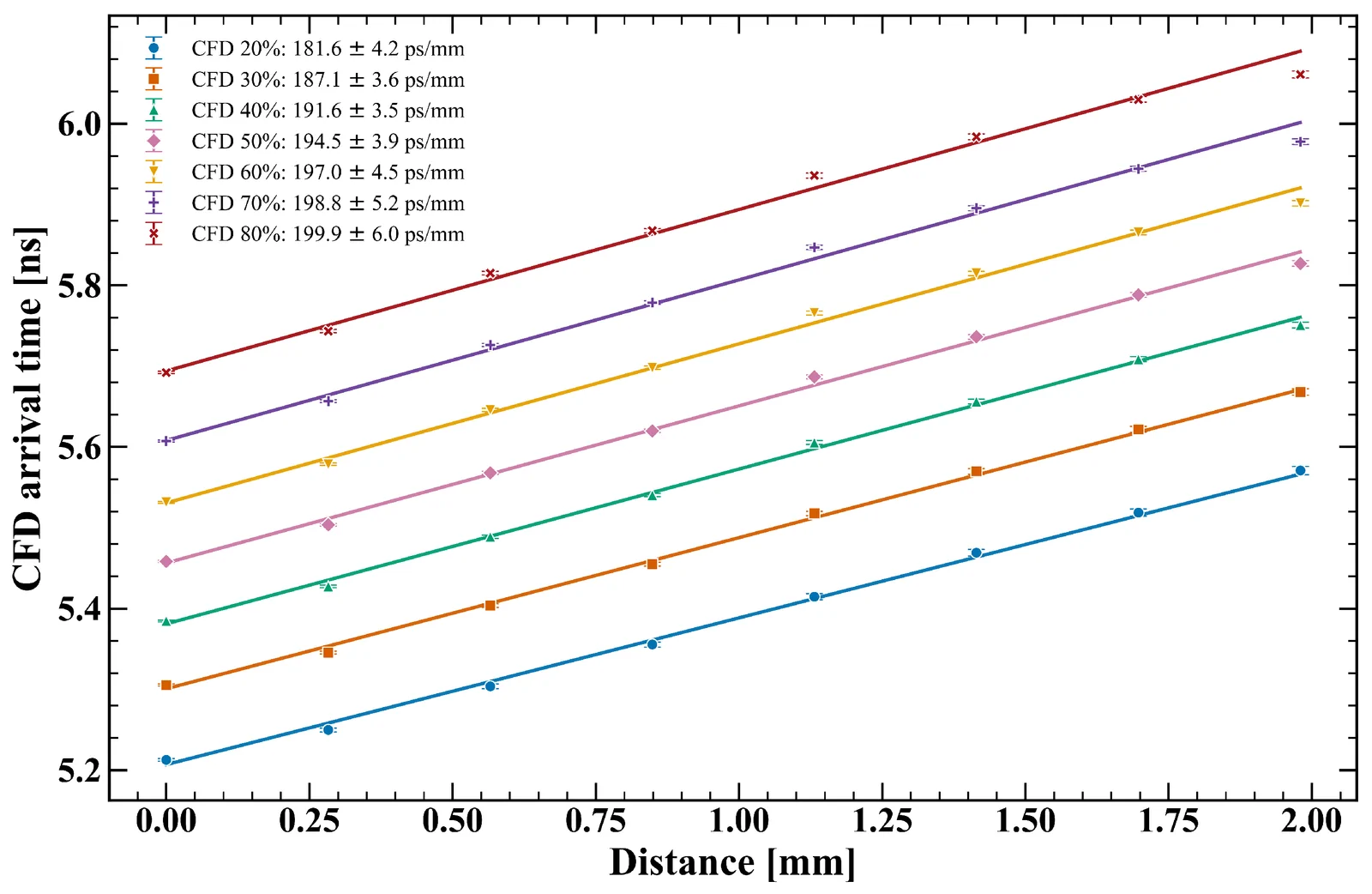
CFD arrival time as a function of effective propagation distance for CFD fractions from 20% to 80%. The points correspond to representative positions along the diagonal scan direction. The data points represent the measured arrival times, and the solid lines show the corresponding linear fits. The moderate increase in the fitted delay slope with CFD fraction is attributed to the slight distance-dependent broadening of the measured waveforms.

To evaluate the dependence on the CFD fraction, the arrival-time analysis was repeated using CFD thresholds from 20% to 80%, as shown in Figure 9. The extracted delay slope increases moderately from 181.6±4.2ps/mm at tCFD0.2 to 199.9±6.0ps/mm at tCFD0.8. This dependence is attributed to the slight broadening of the waveform with increasing propagation distance, which produces a larger position-dependent shift at higher CFD fractions. Nevertheless, the maximum deviation from the tCFD0.4 result is only about 5%, and the approximately linear distance dependence remains unchanged. tCFD0.4 was therefore retained as a representative threshold in themiddle of the leading edge, where the timing extraction is less sensitive to baseline noise at low fractions and waveform distortion near the pulse maximum at high fractions.

### 3.3. Position-Dependent Delay Correction

For an AC-LGAD sensor, the arrival time, measured from the oscilloscope, contains both the intrinsic time response of the sensor and the position-dependent signal propagation delay. Therefore, the corrected arrival time is defined as

(1) $$T_{\mathrm{corrected}}=T_{\mathrm{meas}}-T_{\mathrm{delay}},$$

where $T_{\mathrm{meas}}$ is the arrival time directly extracted from the waveform, and $T_{\mathrm{delay}}$ is the propagation delay associated with the laser injection position. For AC-LGAD sensors, the position-dependent signal propagation delay can be studied using laser-scan measurements. A two-dimensional map of the mean arrival time can be obtained. Since AC-LGAD sensors provide hit-position information, the propagation delay can be extracted by matching the reconstructed hit position to the corresponding mean-arrival-time map [10].

**Figure 10:**
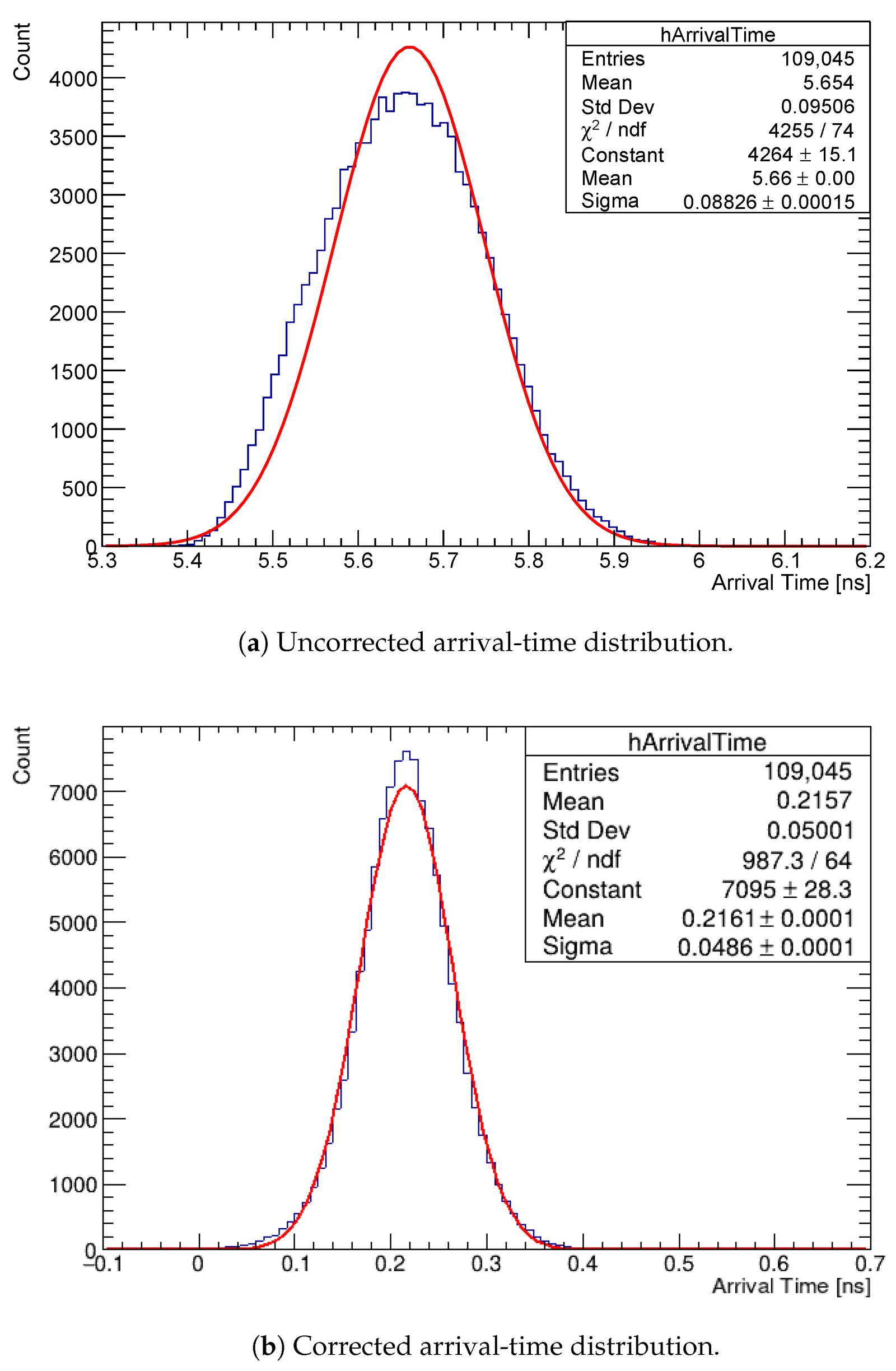
Arrival-time distributions obtained from the same 15×15 laser scan points: (**a**) without correcting for the position-dependent propagation delay, the distribution has a sigma of 88.3ps; (**b**) after applying the position-dependent propagation-delay correction, the sigma is reduced to 48.6ps. The blue stepped histograms represent the measured arrival-time distributions, whereas the red solid curves represent the corresponding Gaussian fits.

Figure 10a shows the arrival-time distribution obtained from the 15×15 laser scan points before delay correction. A simple event-selection cut was applied to remove a small number of outlier events caused by occasional experimental fluctuations. The signal is read out by the lower-left AC pad and all scan positions are included in this distribution. Due to the non-negligible position-dependent signal propagation delay, the distribution is significantly broadened. The sigma of this distribution is σ=88.3ps.

After correcting for the position-dependent time delay using the laser-hit position information, the arrival-time distribution becomes much narrower, as shown in Figure 10b. The sigma of this distribution is σ=48.6ps. This improvement demonstrates that the position-dependent delay is a major contribution to the measured time spread when all scan positions are combined. Therefore, a detailed investigation and correction of the position-dependent time delay are crucial for improving the timing performance of AC-LGAD sensors.

## 4. Simulation Methods and Results

### 4.1. Two-Dimensional Telegrapher’s Equations

**Figure 11:**
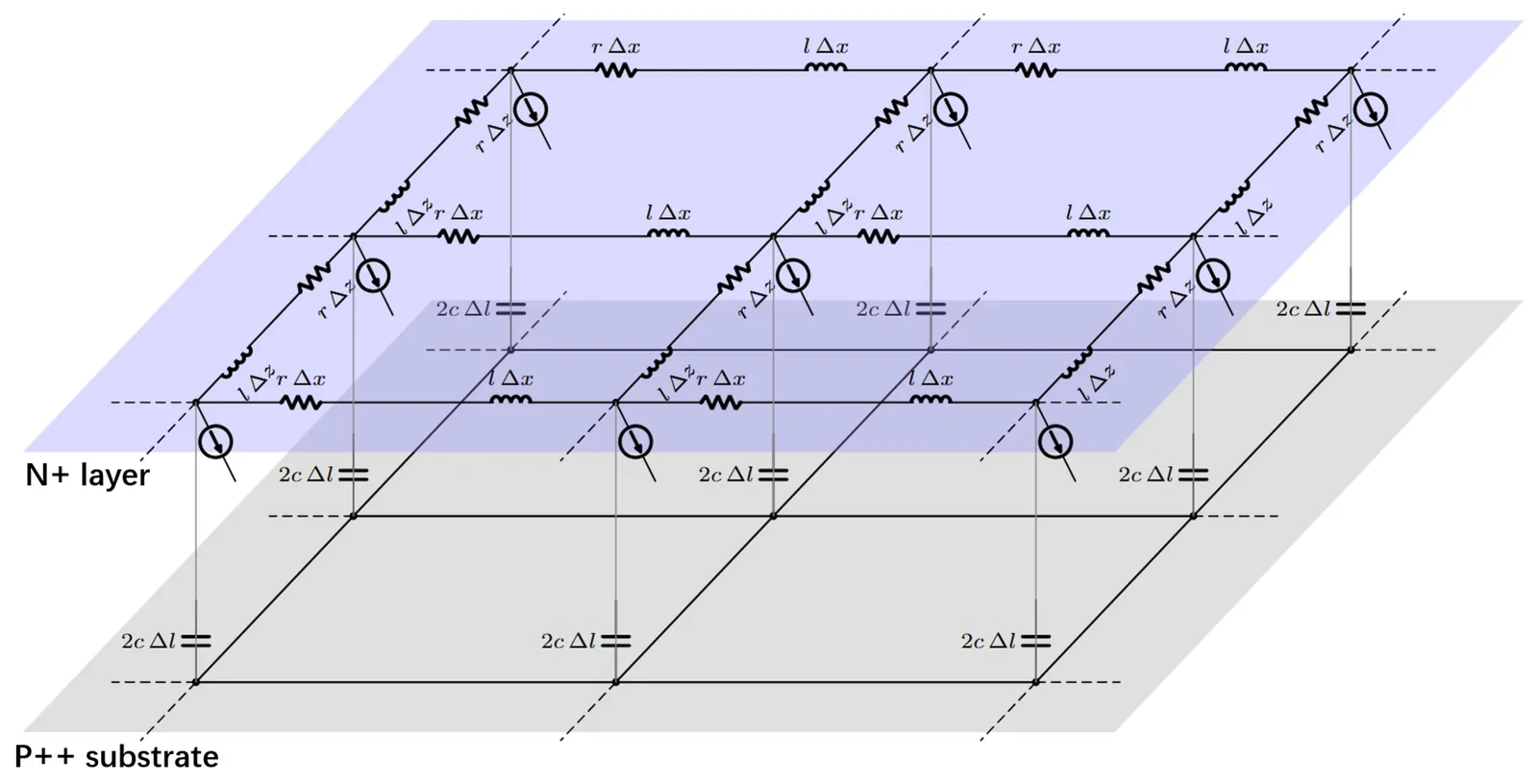
Equivalent circuit mesh of a two-dimensional distributed-parameter transmission line.

For AC-LGADs, the continuous resistive $N^{+}$ layer provides a two-dimensional path for lateral signal transport. The signal therefore does not propagate along a single fixed route, but spreads over the resistive plane and is collected by the surrounding readout electrodes. To describe this process, the AC-LGAD sensor can be modeled as a two-dimensional distributed transmission-line network, as shown in Figure 11. In this model, per-unit-length series resistance and inductance introduced by the resistive layer are denoted by r[Ω/m] and l[H/m], respectively. The distributed capacitance from the node to the reference plane is described by c[F/m] [14,15].

The node voltage is denoted by V(x,z,t), and the branch currents along the *x* and *z* directions are denoted by $I_{x}\left(x,z,t\right)$ and $I_{z}\left(x,z,t\right)$, respectively. By applying Kirchhoff’s current law (KCL) to the nodes and Kirchhoff’s voltage law (KVL) to the infinitesimal branches along the *x* and *z* directions, the following two-dimensional lossy transmission-line equations can be obtained in the continuum limit for a uniform mesh with Δx=Δz=Δl:

(2) $$\begin{array}{cc} \frac{\partial V(x,z,t)}{\partial t} & =-\frac{1}{2c}\left(\frac{\partial I_{x}\left(x,z,t\right)}{\partial x}+\frac{\partial I_{z}\left(x,z,t\right)}{\partial z}\right),\\ \frac{\partial I_{x}\left(x,z,t\right)}{\partial t} & =-\frac{1}{l}\frac{\partial V(x,z,t)}{\partial x}-\frac{r}{l}I_{x}\left(x,z,t\right),\\ \frac{\partial I_{z}\left(x,z,t\right)}{\partial t} & =-\frac{1}{l}\frac{\partial V(x,z,t)}{\partial z}-\frac{r}{l}I_{z}\left(x,z,t\right). \end{array}$$

By differentiating the current equations with respect to the corresponding spatial coordinates and eliminating the current variables, a second-order voltage equation can be obtained:

(3) $$\frac{\partial^{2}V\left(x,z,t\right)}{\partial x^{2}}+\frac{\partial^{2}V\left(x,z,t\right)}{\partial z^{2}}=2cl\frac{\partial^{2}V\left(x,z,t\right)}{\partial t^{2}}+2cr\frac{\partial V(x,z,t)}{\partial t}.$$

This equation is the two-dimensional lossy telegraph-type equation. In the frequency domain, assuming the time convention $e^{j\omega t}$, Equation (3) becomes

(4) $$\left(\frac{\partial^{2}}{\partial x^{2}}+\frac{\partial^{2}}{\partial z^{2}}\right)V\left(x,z,\omega \right)=\left(-2cl\omega^{2}+j2cr\omega \right)V\left(x,z,\omega \right).$$

Taking a plane-wave solution in the two-dimensional plane,

(5) $$V\left(x,z,\omega \right)\propto \exp\left[-jk\;\cdot \;r\right],$$

one has

(6) $$\left(\frac{\partial^{2}}{\partial x^{2}}+\frac{\partial^{2}}{\partial z^{2}}\right)V\left(x,z,\omega \right)=-\left(k_{x}^{2}+k_{z}^{2}\right)V\left(x,z,\omega \right).$$

Substituting this expression into Equation (4) gives the two-dimensional dispersion relation

(7) $$k^{2}\equiv k_{x}^{2}+k_{z}^{2}=2cl\omega^{2}-j2cr\omega ,$$

where *k* is the magnitude of the two-dimensional wave vector.

For the AC-LGAD resistive layer, the model parameters can be related to the physical sheet quantities. $R_{s}$ is the sheet resistance of the resistive layer, $L_{s}$ is the sheet inductance, and $C_{A}$ the capacitance per unit area between the resistive layer and the anode [16]. For a square mesh with spacing Δ, the series resistance and inductance of one branch are

(8) $$r\Delta =R_{s},\;l\Delta =L_{s},\;2c=C_{A}\Delta .$$

Therefore,

(9) $$r=\frac{R_{s}}{\Delta },\;l=\frac{L_{s}}{\Delta },\;c=\frac{C_{A}\Delta }{2}.$$

Substituting these relations into Equation (7), one obtains

(10) $$k_{x}^{2}+k_{z}^{2}=L_{s}C_{A}\omega^{2}-jR_{s}C_{A}\omega =-\left(R_{s}+j\omega L_{s}\right)\times j\omega C_{A}.$$

This equation represents the dispersion relation for the signal transmission process in the resistive layer of AC-LGAD. It indicates that different combinations of sheet resistance, sheet inductance, and coupling capacitance can lead to different signal propagation characteristics. The detailed step-by-step derivation is provided in Appendix A.

### 4.2. Equivalent Signal Model

The voltage waveform measured by the oscilloscope is not the raw avalanche current generated inside the detector. Instead, it can be expressed as the convolution of three contributions: the source signal generated by avalanche multiplication, the lateral transport response in the resistive layer, and the common electronics response,

(11) $$V_{\mathrm{osc}}\left(t\right)=I_{\mathrm{src}}\left(t\right)⊗H_{\mathrm{tr}}\left(d,t\right)⊗H_{\mathrm{elec}}\left(t\right),$$

where *d* is the propagation distance in the resistive layer.

The transport term is described by the lossy transmission-line model introduced in Section 4.1.

The common electronics response is modeled as one high-pass stage followed by two low-pass stages,

(12) $${\tilde{H}}_{\mathrm{elec}}\left(\omega \right)=\frac{j\omega \tau_{\mathrm{hp}}}{1+j\omega \tau_{\mathrm{hp}}}\;\cdot \;\frac{1}{1+j\omega \tau_{\mathrm{lp}1}}\;\cdot \;\frac{1}{1+j\omega \tau_{\mathrm{lp}2}}.$$

Using the waveform measured near the reference point, the source signal and electronics parameters were extracted, and the waveform at larger distances was calculated by re-convolving the source pulse with the transport response and the electronics transfer function.

### 4.3. Waveform Fit with the Transmission-Line Model

**Figure 12:**
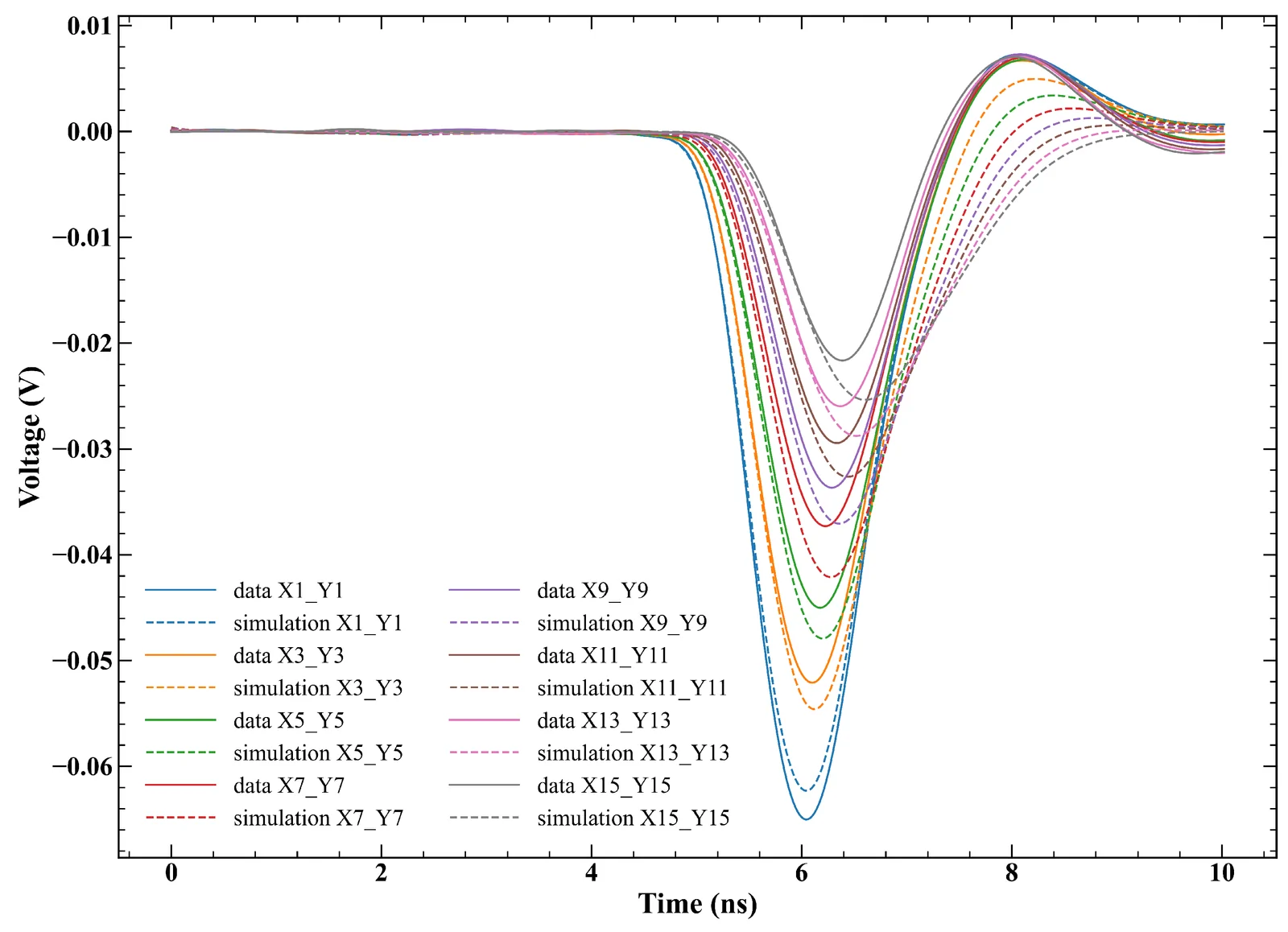
Comparison between measured waveforms (solid lines) and simulated waveforms (dashed lines) for representative points along the diagonal scan direction. The transmission-line model reproduces the leading-edge delay well.

A comparison between the measured and simulated waveforms is shown in Figure 12. The fit was obtained with a sheet resistance of about 80Ω/sq, an areal capacitance of about $4\;\mathrm{pF}/{\mathrm{mm}}^{2}$, and a sheet inductance of about 33pH/sq. The areal capacitance was measured by C-V test, and the sheet inductance was estimated based on the propagation velocity of electromagnetic waves in silicon. The sheet resistance was used as an adjustable parameter in the simulation and was taken to be about 80Ω/sq. This value is reasonably close to the measured sheet resistance of the $N^{+}$ layer, 91–95Ω/sq. The leading edge of the simulated waveform agrees well with the measurement. Since the signal arrival time is typically determined from the threshold-crossing time on the leading edge, this agreement is essential for accurately describing the position-dependent propagation delay.

The slight amplitude difference between the simulated and measured waveforms can be attributed to charge sharing among multiple readout channels, which modifies the measured peak amplitude. The trailing-edge discrepancy is likely caused by the common readout electronics, which is only approximately described by the simplified electronics model used in this work. This simplified model does not fully reproduce the preamplifier, amplifier, cables, and oscilloscope responses. It therefore cannot fully describe the complete waveform, particularly its trailing edge.

**Figure 13:**
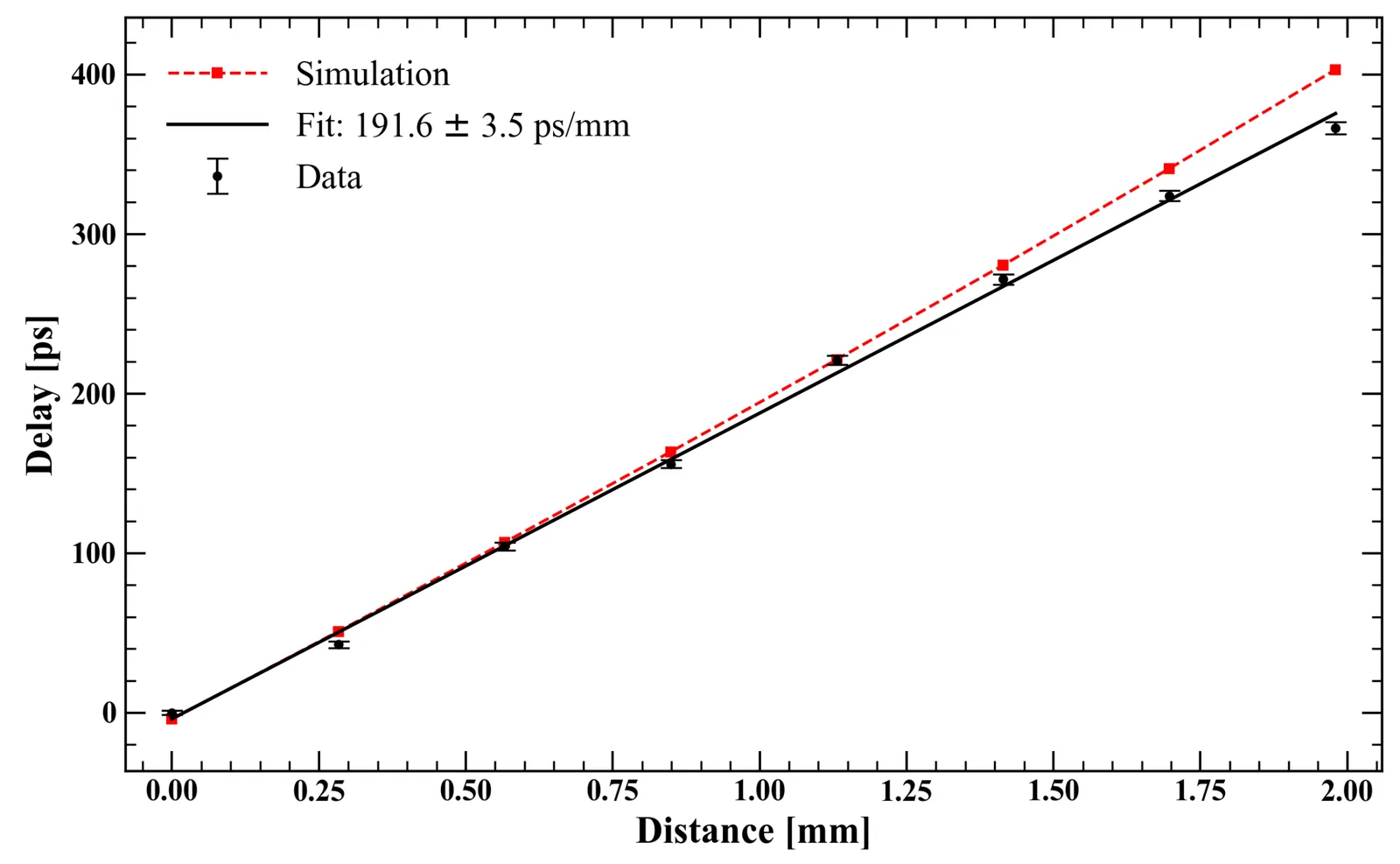
Comparison between measured tCFD0.4 (solid lines) and simulated tCFD0.4 (dashed lines) for representative points along the diagonal scan direction.

The position-dependent delay is successfully reproduced, as shown by the comparison between the measured and simulated delays in Figure 13. The trailing edge is less well modeled, which is attributed mainly to the simplified description of the common electronics response. In addition, the model used here does not explicitly include the detailed multi-pad charge-sharing process, so some discrepancy in the absolute amplitude remains.

**Figure 14:**
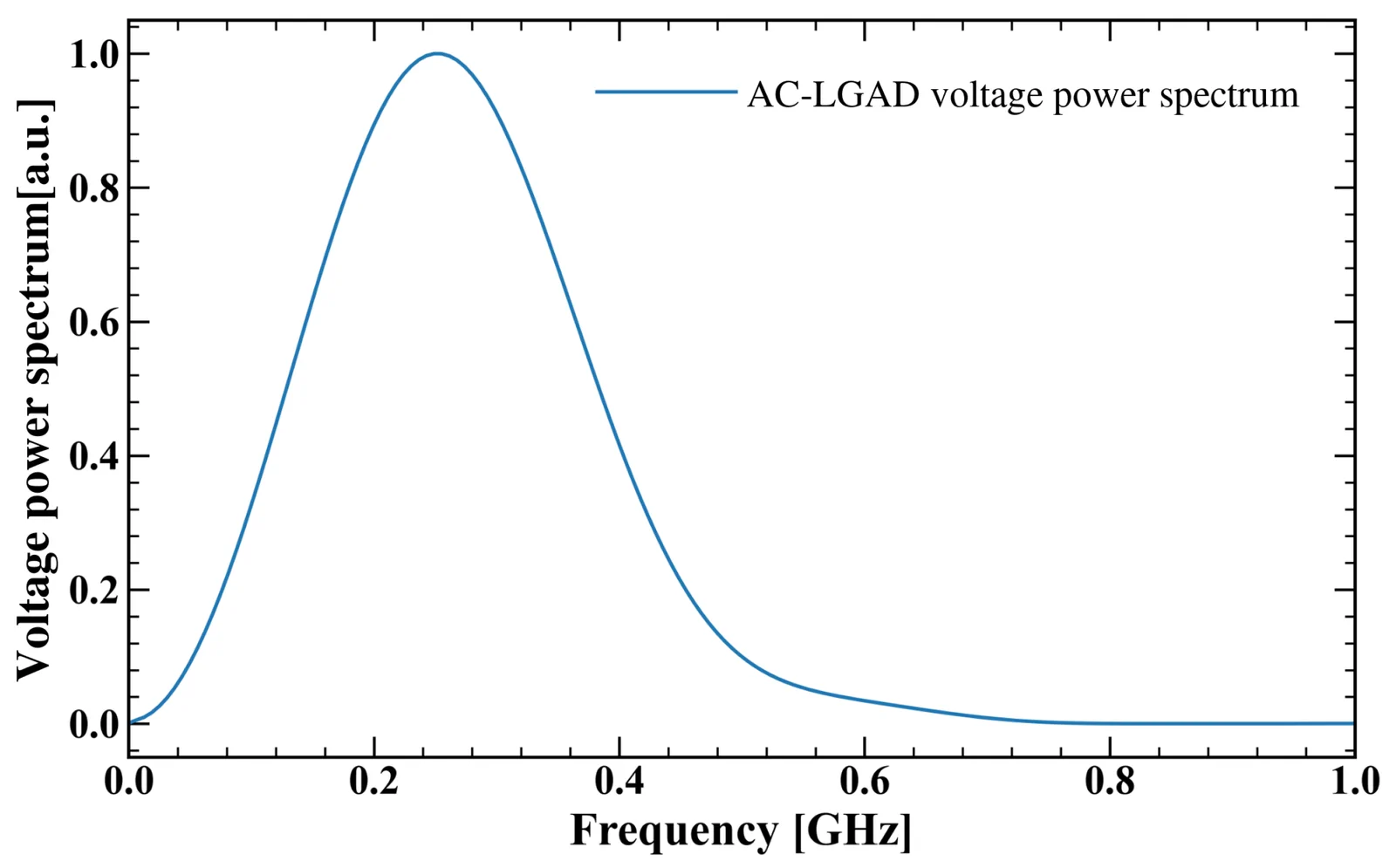
The voltage power spectrum of the AC-LGAD signal is mainly concentrated below approximately 1 GHz.

The frequency-domain voltage power spectrum of the experimentally measured waveform is shown in Figure 14. It was obtained by applying a one-sided fast Fourier transform (FFT) to the baseline-subtracted time-domain voltage waveform, V(t), and then calculating the squared magnitude of the Fourier coefficients,

(13) $$P\left(f\right)\propto {\left|\tilde{V}\left(f\right)\right|}^{2},\;\tilde{V}\left(f\right)=\mathrm{FFT}\left[V\left(t\right)\right].$$

The spectrum was normalized to its maximum value. The resulting spectrum represents the relative distribution of the measured signal energy over frequency. The voltage power spectrum of the AC-LGAD signal is mainly concentrated below approximately 1 GHz. In this frequency range, the resistive term dominates over the inductive term in the propagation constant, so the transport is closer to a diffusion-like regime than to a low-loss wave-propagation regime. Nevertheless, the dominant spectral components are sufficiently concentrated that, over the short measured distance, the delay can still be approximated by a linear dependence on distance. This explains the substantial delay, dispersion, and amplitude attenuation observed in the waveforms.

**Figure 15:**
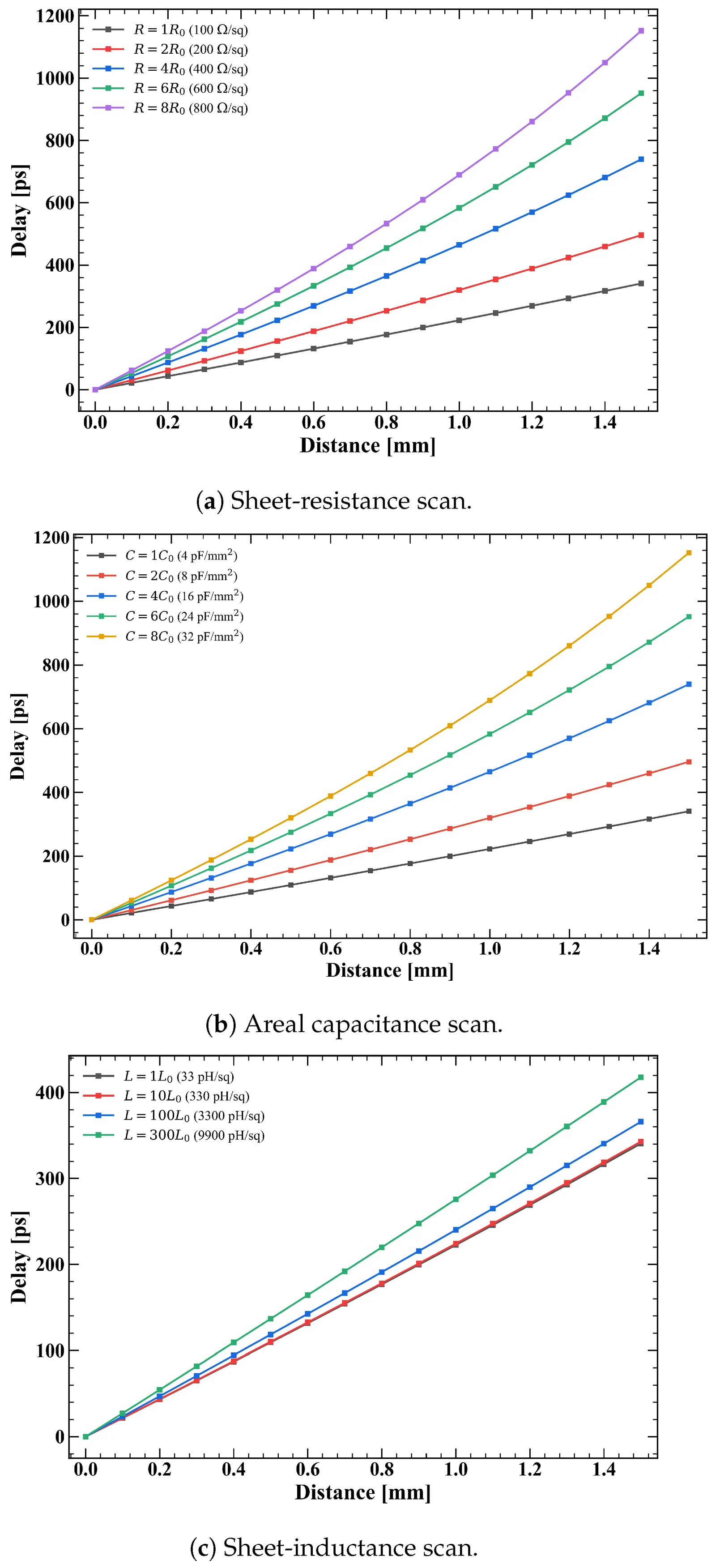
Simulated tCFD0.4 delay as a function of propagation distance for different transmission-line parameters: (**a**) sheet resistance with $C_{A}=C_{0}$ and $L_{\mathrm{sheet}}=L_{0}$; (**b**) areal capacitance with $R_{\mathrm{sheet}}=R_{0}$ and $L_{\mathrm{sheet}}=L_{0}$; and (**c**) sheet inductance with $R_{\mathrm{sheet}}=R_{0}$ and $C_{A}=C_{0}$.

To evaluate the relative contributions of the transmission-line parameters to the position-dependent delay, three parameter scans were performed using the nominal values $R_{0}=100\;\Omega /\mathrm{sq}$, $C_{0}=4\;\mathrm{pF}/{\mathrm{mm}}^{2}$, and $L_{0}=33\;\mathrm{pH}/\mathrm{sq}$. The extracted slopes of delay versus propagation distance are summarized in Table 1, and the corresponding delay curves are shown in Figure 15. When $C_{A}$ and $L_{\mathrm{sheet}}$ are fixed at their nominal values, increasing $R_{\mathrm{sheet}}$ from 100 to 800Ω/sq increases the delay slope from 219 to 640ps/mm.

Similarly, when $R_{\mathrm{sheet}}$ and $L_{\mathrm{sheet}}$ are fixed, increasing $C_{A}$ from 4 to $32\;\mathrm{pF}/{\mathrm{mm}}^{2}$ produces the same delay-slope increase as the corresponding sheet-resistance scan, from 219 to 640ps/mm. In contrast, increasing $L_{\mathrm{sheet}}$ alone by a factor of 300, from 33 to 9900pH/sq, increases the delay slope only from 219 to 273ps/mm. Within the present effective model, these results indicate that the propagation delay is governed primarily by the resistive–capacitive term, whereas the sheet inductance has a comparatively weak influence.

### 4.4. Distributed SPICE Network Cross-Check

To provide a complementary circuit-level cross-check of the continuum transmission-line model, the active sensor region and the four-pad readout geometry were implemented as a distributed RLC network in LTspice (v26.0.1). The $6\times 6\;{\mathrm{mm}}^{2}$ sensor was discretized into a 61×61 node grid with a spatial interval of Δ=100μm. Adjacent nodes in the continuous N^+^ layer were connected through a series resistance and inductance. Since each branch represents one square of the resistive layer, the branch parameters were set to $R_{\mathrm{branch}}=R_{\mathrm{sheet}}=80\;\Omega$ and $L_{\mathrm{branch}}=L_{\mathrm{sheet}}=33\;\mathrm{pH}$.

Each grid cell was connected to the reference plane through a distributed capacitance with an areal density of $C_{A}=4\;\mathrm{pF}/{\mathrm{mm}}^{2}$. In the regions covered by the four $1.5\times 1.5\;{\mathrm{mm}}^{2}$ readout pads, an additional oxide capacitance density of $C_{\mathrm{ox}}=288\;\mathrm{pF}/{\mathrm{mm}}^{2}$ was included between the N^+^ layer and the corresponding metal pad.

The effective source-current waveform was extracted using the measured waveform at the reference position X1Y1 together with the corresponding SPICE transfer response. Regularized deconvolution was applied to remove the local sensor-network response, and the reconstructed time-domain current was then implemented as a PWL current source in LTspice. The same effective source waveform was used for all investigated injection positions along the diagonal direction.

**Figure 16:**
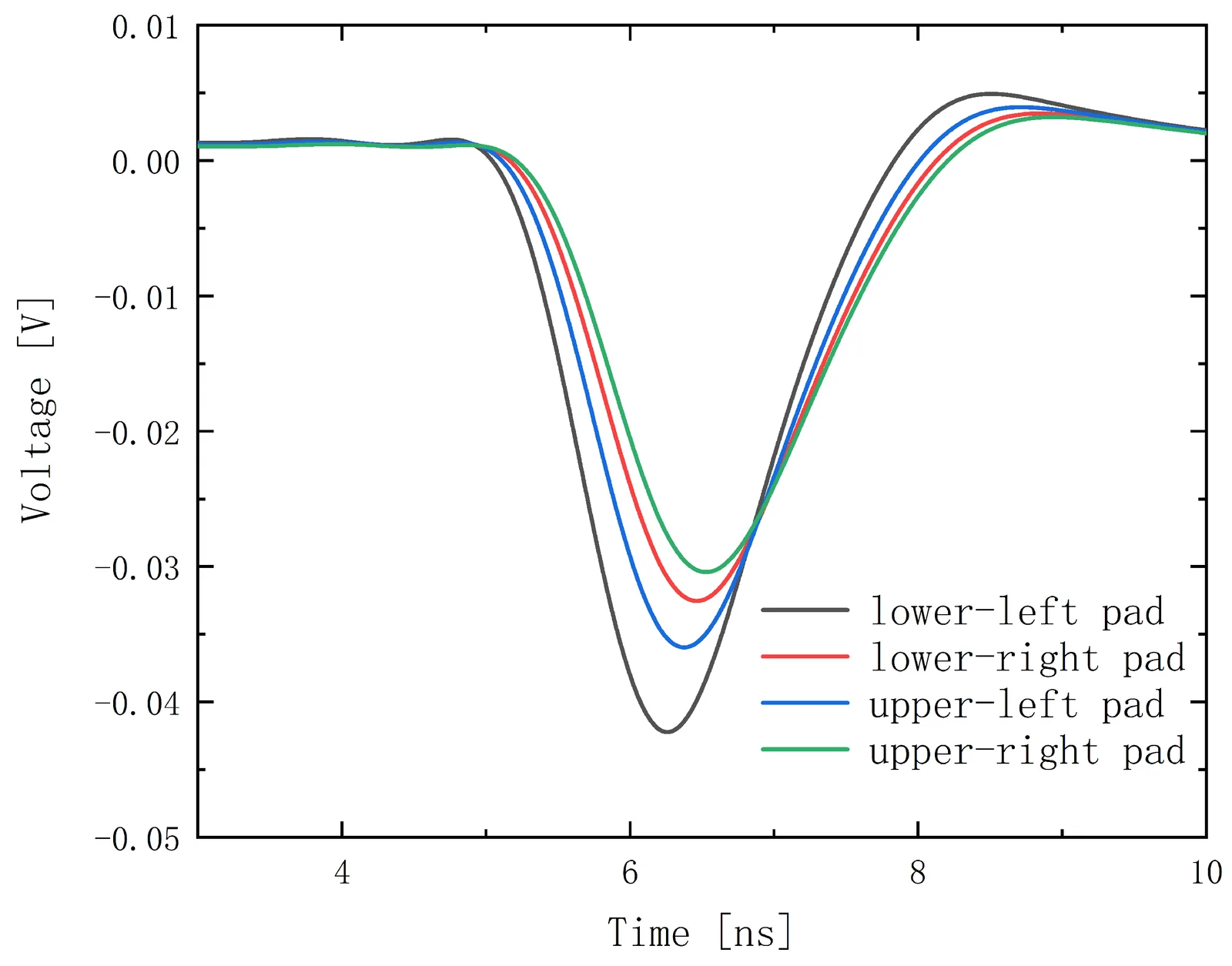
SPICE-simulated voltage waveforms of the four AC readout pads for a laser-equivalent current injection at X4Y6. The differences in pulse amplitude and arrival time result from the position-dependent lateral signal transport and capacitive charge sharing in the distributed RLC network.

Figure 16 shows the simulated signals induced on all four pads for an injection at X4Y6. The lower-left pad, which is closest to the injection position, exhibits the largest and earliest response. The signals on the other pads become progressively smaller and later with increasing distance from the injection point. Thus, in contrast to the simplified analytical signal model, the distributed SPICE network explicitly includes the multi-pad capacitive charge-sharing process.

**Figure 17:**
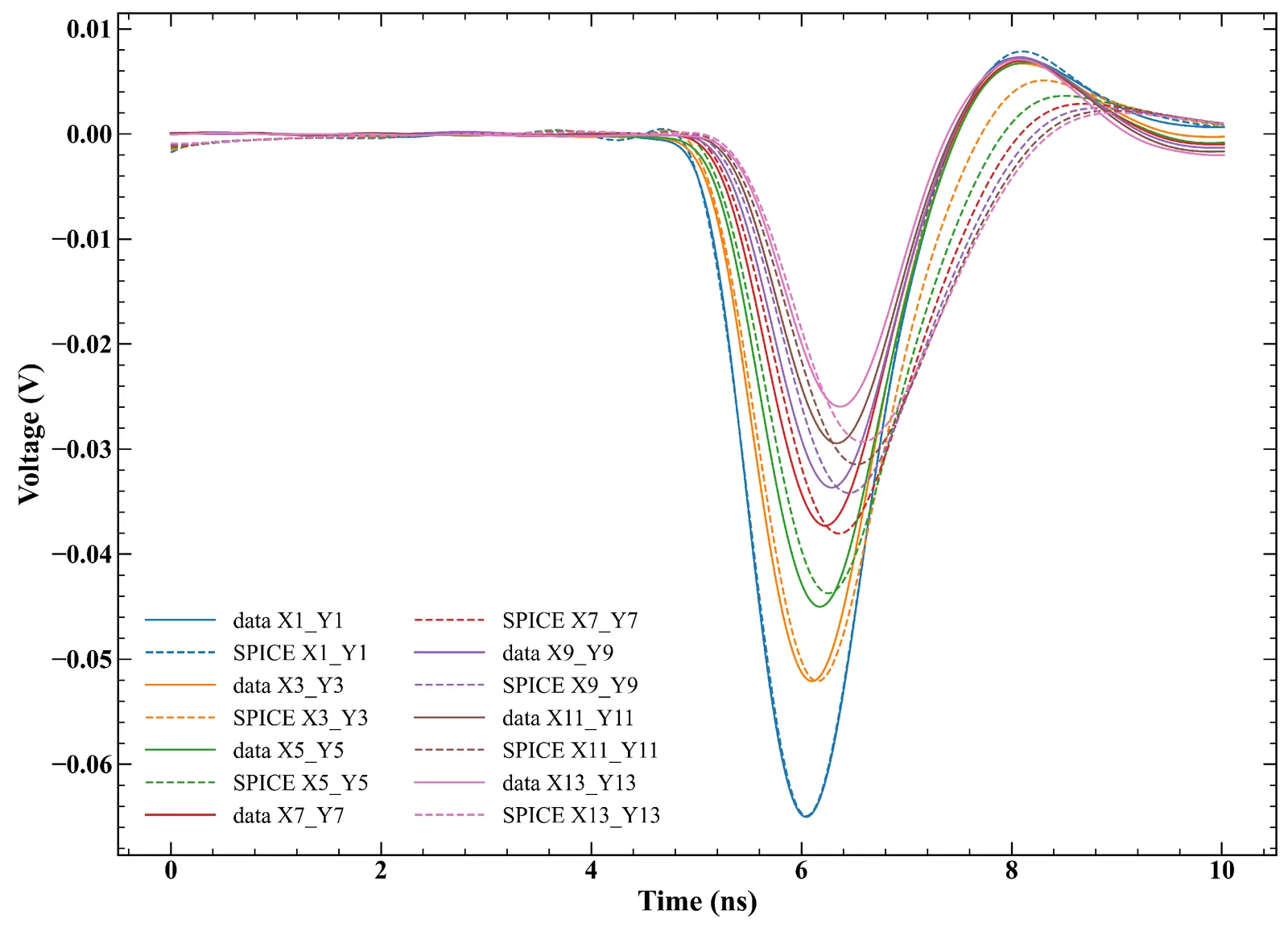
Comparison between the measured waveforms (solid lines) and the corresponding SPICE-simulated waveforms (dashed lines) at representative positions along the diagonal scan direction. The same color denotes the experimental and SPICE waveforms at the same injection position.

Figure 17 compares the measured and SPICE-simulated waveforms at representative positions along the diagonal scan direction. The SPICE simulation reproduces the leading-edge evolution consistently with the preceding two-dimensional transmission-line model. By explicitly including the capacitive coupling to multiple pads, it also provides a more complete description of charge sharing and the relative signal amplitudes. Nevertheless, the agreement gradually deteriorates at larger propagation distances, indicating limitations in the prediction of the complete waveform.

**Figure 18:**
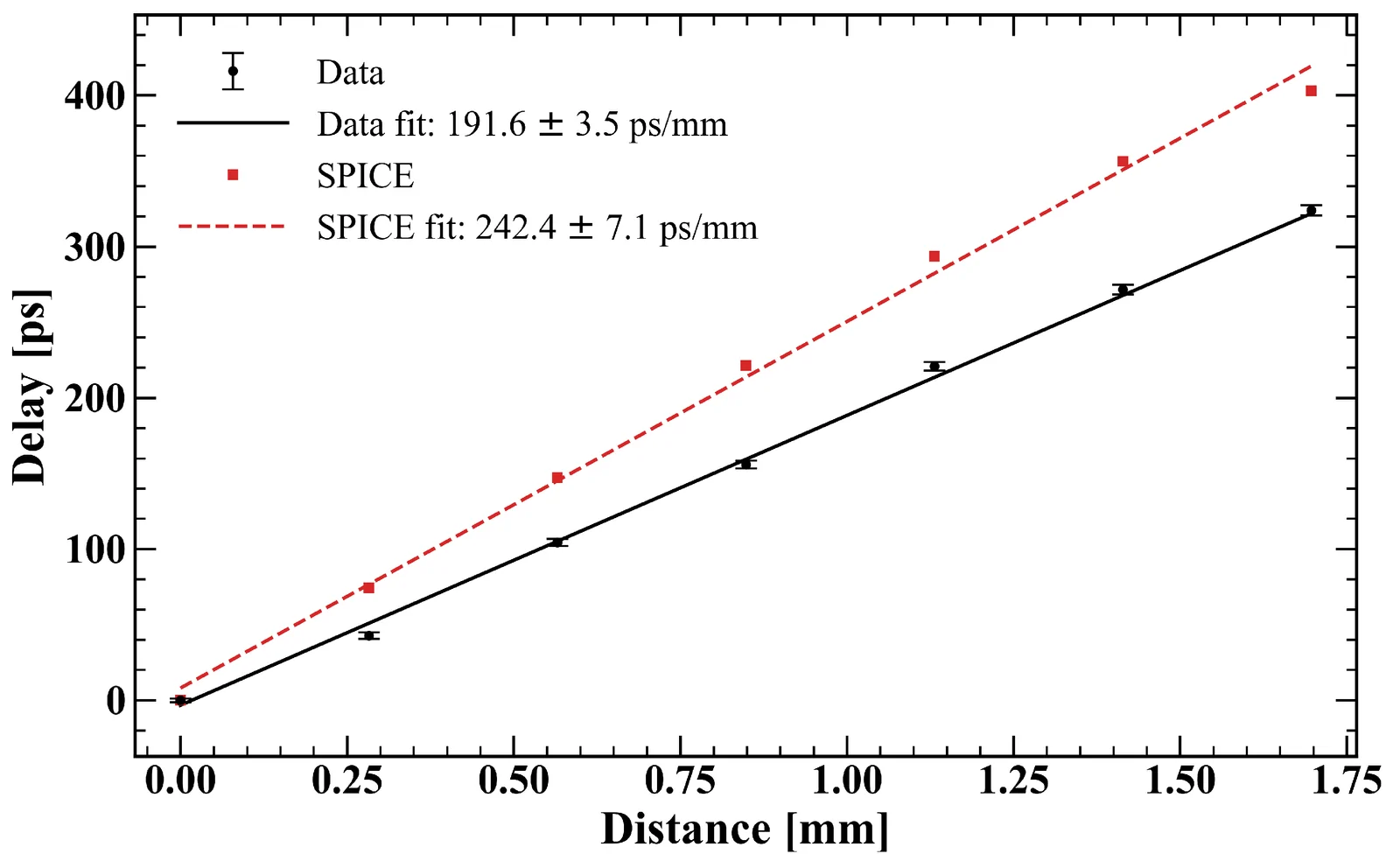
Relative tCFD0.4 delay as a function of the effective propagation distance for the experimental data and the distributed SPICE simulation. The delays are referenced to X1Y1, and the solid and dashed lines represent linear fits to the experimental and SPICE results, respectively. The fitted slopes are 191.6±3.5ps/mm for the experimental data and 242.4±7.1ps/mm for the SPICE simulation.

The relative tCFD0.4 delays extracted from the diagonal waveforms are shown in Figure 18. The distributed SPICE network gives a delay slope of 242.4±7.1ps/mm, approximately 26.5% larger than the measured value of 191.6±3.5ps/mm. Although the quantitative agreement is less close than that of the effective two-dimensional transmission-line model, the SPICE simulation reproduces the approximately linear distance dependence and provides a complementary circuit-level cross-check of the relationship between the propagation delay and the distributed RLC properties of the device.

Unlike the preceding effective model, in which the signal propagation was parameterized by the shortest distance to the pad edge, the SPICE pad output is obtained from the combined contributions of all grid nodes capacitively coupled to the pad area. The propagation distances associated with these contributions are different and therefore cannot be represented exactly by a single effective distance. In addition, the complex back-end electronics are not fully included, and residual readout response may remain in the effective source waveform after regularized deconvolution. Therefore, the SPICE results are interpreted as a complementary circuit-level cross-check of the two-dimensional transmission-line model rather than a complete reproduction of the measured waveforms.

## 5. Discussion

### 5.1. Influence of Source-Current Characteristics on the Measured Propagation Delay

In the equivalent signal model, the avalanche-generated source current is an important input. Although the distributed transfer response is determined by the electrical parameters and geometry of the sensor, its dispersion relation is frequency-dependent, as shown in Section 4.1. Consequently, the bandwidth and spectral shape of the source current affect the propagated waveform and may influence the CFD-derived delay. The source-current waveform is determined mainly by the active sensor thickness, bias voltage, gain-layer structure, and excitation source [17,18,19].

The bias voltage controls the internal electric field, carrier drift velocity, and multiplication gain. The gain-layer thickness, depth, and doping profile further influence the local high-field distribution and avalanche multiplication, thereby affecting the amplitude, rise time, and spectral content of the source-current pulse. As the relevant carrier velocities approach saturation, the bulk transit time decreases and the leading edge becomes faster; further increases in bias then have a reduced effect on the bulk transit time, although the gain may continue to change. For planar LGADs operated near velocity saturation and under otherwise comparable conditions, the signal slew rate approximately follows [17]

(14) $$\frac{dV}{dt}\propto \frac{G}{d},$$

where *G* is the multiplication gain and *d* is the active thickness. Thus, gain-layer-dependent variations in *G* primarily modify the signal amplitude and absolute slew rate, whereas a smaller active thickness and shorter carrier transit time produce a faster leading edge.

Laser- and MIP-induced signals differ primarily in the formation and fluctuations of the source current. The absorption length of 1064 nm light in silicon is of the order of 1 mm, much larger than the 50 μm sensor thickness, resulting in approximately uniform carrier generation along the depth. In contrast, MIPs produce discrete ionization clusters and event-to-event fluctuations in the deposited charge and induced-current shape [20,21]. Consequently, the detailed waveform and absolute CFD-derived delay slope may differ between laser and particle measurements, and the magnitude of this difference is not quantified in the present study.

Nevertheless, the device structure, bias voltage, and excitation condition were all kept fixed throughout the spatial scan. Therefore, the systematic variation with propagation distance originates from the position-dependent transfer response of the distributed sensor network. Source-current characteristics, including those associated with the gain-layer design and excitation mechanism, may modify the numerical value of the extracted delay slope, but they do not change the underlying RLC-controlled propagation mechanism. Because only one gain-layer design and one bias condition were investigated, their individual quantitative contributions cannot be isolated in the present study. Accordingly, these limitations do not affect the principal conclusion that the observed position dependence is governed primarily by the distributed electrical properties of the resistive layer.

### 5.2. Applicability and Limitations of the Effective Propagation-Distance Approximation

The signal measured by an AC pad represents the spatially integrated response over the electrode area. Similarly, in the distributed SPICE model, the pad waveform is obtained by summing the responses of all nodes capacitively coupled to the same electrode. The equivalent signal model simplifies this process by using the shortest distance between the injection position and the pad, assuming that the leading edge is dominated by the nearest electrode region. For the compact square pixel investigated here, the overall distribution of propagation paths is strongly correlated with this minimum distance. Therefore, the experiment, equivalent model, and distributed SPICE calculation all exhibit an approximately linear delay–distance relationship.

This approximation is geometry-dependent. For elongated, non-convex, or cross-shaped electrodes, the same minimum distance may correspond to different distributions of propagation paths, making a single effective distance insufficient. Nevertheless, a complex electrode can be divided computationally into multiple small and locally compact components. The equivalent signal model remains applicable to each component using its individual propagation distance and geometrical weighting. The total electrode waveform can then be obtained by summing the waveform contributions from all components before extracting the CFD arrival time. Therefore, the model can be extended to more complex electrode geometries through geometrical discretization and waveform summation.

## 6. Conclusions

Position-dependent signal propagation in a large-pitch pixel AC-LGAD was investigated using a two-dimensional picosecond-laser scan and four-channel waveform readout. For the tested device, the leading-edge arrival time shows a clear dependence on the laser injection position. When the delay is parameterized by the effective propagation distance, an approximately linear dependence is observed over the scanned region, with an extracted delay slope of 194.7±1.3ps/mm. After applying the position-dependent delay correction, the sigma of the combined arrival-time distribution over the scanned region is reduced from 88.3ps to 48.6ps.

The measured leading-edge delay can be described by an effective two-dimensional lossy transmission-line model of the continuous resistive layer. The model provides a semi-quantitative interpretation of the observed position-dependent delay and indicates that, within the measured signal bandwidth, the resistive term dominates over the inductive term. The lateral signal transport is therefore dispersive and diffusion-like, while the near-linear CFD delay observed in the finite scan range can be treated as an effective leading-edge timing behavior. A distributed SPICE network that explicitly includes multi-pad capacitive charge sharing reproduces the approximately linear distance dependence and the relative signal-amplitude and arrival-time ordering. The result provides a complementary circuit-level cross-check of the distributed-RLC interpretation.

A scan of the resistive-layer sheet resistance shows that the delay slope increases significantly with increasing $R_{\mathrm{sheet}}$, indicating that the sheet resistance is a key design parameter for balancing charge sharing, signal attenuation, and timing uniformity. In contrast, the scan of the resistive-layer sheet inductance shows that increasing $L_{\mathrm{sheet}}$ produces only a minor change in the delay slope, indicating that the sheet inductance has a comparatively weak influence within the relevant signal bandwidth. The position-dependent delay may therefore be reduced by moderately decreasing the sheet resistance and effective capacitive load, or by shortening the propagation distance through a smaller pad pitch or larger pad fill factor. These results provide useful guidance for position-dependent timing correction and future optimization of AC-LGAD sensor design.

## Figures and Tables

**Table 1:** Transmission-line parameter scans and simulated delay slopes.

$R_{\mathrm{sheet}}$ (Ω/sq)	$L_{\mathrm{sheet}}$ (pH/sq)	$C_{A}$ (pF/mm^2^)	Delay Slope (ps/mm)
100	33	4.0	219
100	33	8.0	311
100	33	16.0	445
100	33	24.0	550
100	33	32.0	640
200	33	4.0	311
400	33	4.0	445
600	33	4.0	550
800	33	4.0	640
100	330	4.0	220
100	3300	4.0	237
100	9900	4.0	273

## Data Availability

The original data presented in this study are openly available in Zenodo at: https://doi.org/10.5281/zenodo.20697659.

## Appendix A. Derivation of the Two-Dimensional Lossy Transmission-Line Equations

The resistive layer is represented by a uniform square mesh with spatial intervals Δx=Δz=Δ. The voltage at the node (i,j) is denoted by $V_{i,j}\left(t\right)$. The currents flowing in the positive *x* and *z* directions are denoted by $I_{x,i+1/2,j}\left(t\right)$ and $I_{z,i,j+1/2}\left(t\right)$, respectively. Each branch contains a series resistance rΔ and a series inductance lΔ, while the shunt capacitance associated with each node is 2cΔ.

Applying Kirchhoff’s current law to node (i,j) gives

(A1) $$\begin{array}{cc} 2c\Delta \frac{dV_{i,j}}{dt}= & I_{x,i-1/2,j}-I_{x,i+1/2,j}\\ \; & +I_{z,i,j-1/2}-I_{z,i,j+1/2}. \end{array}$$

Dividing Equation (A1) by Δ and taking the continuum limit Δ→0 yields

(A2) $$2c\frac{\partial V}{\partial t}=-\frac{\partial I_{x}}{\partial x}-\frac{\partial I_{z}}{\partial z},$$

or equivalently,

(A3) $$\frac{\partial V}{\partial t}=-\frac{1}{2c}\left(\frac{\partial I_{x}}{\partial x}+\frac{\partial I_{z}}{\partial z}\right).$$

For a branch connecting the nodes (i,j) and (i+1,j), Kirchhoff’s voltage law gives

(A4) $$V_{i,j}-V_{i+1,j}=r\Delta I_{x,i+1/2,j}+l\Delta \frac{dI_{x,i+1/2,j}}{dt}.$$

Dividing by Δ and taking the continuum limit gives

(A5) $$\frac{\partial V}{\partial x}=-rI_{x}-l\frac{\partial I_{x}}{\partial t},$$

and therefore

(A6) $$\frac{\partial I_{x}}{\partial t}=-\frac{1}{l}\frac{\partial V}{\partial x}-\frac{r}{l}I_{x}.$$

Similarly, applying KVL to a branch in the *z* direction gives

(A7) $$\frac{\partial I_{z}}{\partial t}=-\frac{1}{l}\frac{\partial V}{\partial z}-\frac{r}{l}I_{z}.$$

Equations (A3), (A6) and (A7) constitute the first-order two-dimensional lossy transmission-line equations:

(A8) $$\begin{array}{cc} \frac{\partial V}{\partial t} & =-\frac{1}{2c}\left(\frac{\partial I_{x}}{\partial x}+\frac{\partial I_{z}}{\partial z}\right),\\ \frac{\partial I_{x}}{\partial t} & =-\frac{1}{l}\frac{\partial V}{\partial x}-\frac{r}{l}I_{x},\\ \frac{\partial I_{z}}{\partial t} & =-\frac{1}{l}\frac{\partial V}{\partial z}-\frac{r}{l}I_{z}. \end{array}$$

To eliminate the current variables, Equations (A6) and (A7) are differentiated with respect to *x* and *z*, respectively:

(A9) $$\frac{\partial }{\partial t}\left(\frac{\partial I_{x}}{\partial x}+\frac{\partial I_{z}}{\partial z}\right)=-\frac{1}{l}\left(\frac{\partial^{2}V}{\partial x^{2}}+\frac{\partial^{2}V}{\partial z^{2}}\right)-\frac{r}{l}\left(\frac{\partial I_{x}}{\partial x}+\frac{\partial I_{z}}{\partial z}\right).$$

According to Equation (A2),

(A10) $$\frac{\partial I_{x}}{\partial x}+\frac{\partial I_{z}}{\partial z}=-2c\frac{\partial V}{\partial t}.$$

Substituting Equation (A10) into Equation (A9) and rearranging gives

(A11) $$\frac{\partial^{2}V}{\partial x^{2}}+\frac{\partial^{2}V}{\partial z^{2}}=2cl\frac{\partial^{2}V}{\partial t^{2}}+2cr\frac{\partial V}{\partial t}.$$

Equation (A11) is a two-dimensional lossy telegraph-type equation. The first term on its right-hand side represents the inductive wave-propagation contribution, whereas the second term represents the resistive diffusion and attenuation contribution.

Using the time convention $e^{j\omega t}$,

(A12) $$\frac{\partial }{\partial t}\to j\omega ,\;\frac{\partial^{2}}{\partial t^{2}}\to -\omega^{2},$$

Equation (A11) becomes

(A13) $$\left(\frac{\partial^{2}}{\partial x^{2}}+\frac{\partial^{2}}{\partial z^{2}}\right)V\left(x,z,\omega \right)=\left(-2cl\omega^{2}+j2cr\omega \right)V\left(x,z,\omega \right).$$

For a plane-wave solution

(A14) $$V\left(x,z,\omega \right)=V_{0}\exp\left[-j(k_{x}x+k_{z}z)\right],$$

the spatial derivatives satisfy

(A15) $$\left(\frac{\partial^{2}}{\partial x^{2}}+\frac{\partial^{2}}{\partial z^{2}}\right)V=-\left(k_{x}^{2}+k_{z}^{2}\right)V.$$

Substitution into Equation (A13) gives

(A16) $$k_{x}^{2}+k_{z}^{2}=2cl\omega^{2}-j2cr\omega .$$

The distributed parameters can be related to the physical sheet parameters of the AC-LGAD. Let $R_{s}$ denote the sheet resistance, $L_{s}$ the sheet inductance, and $C_{A}$ the capacitance per unit area between the resistive layer and the anode [16]. For a square mesh with spacing Δ, the branch resistance, branch inductance, and nodal capacitance are

(A17) $$r\Delta =R_{s},\;l\Delta =L_{s},\;2c\Delta =C_{A}\Delta^{2}.$$

Consequently,

(A18) $$r=\frac{R_{s}}{\Delta },\;l=\frac{L_{s}}{\Delta },\;c=\frac{C_{A}\Delta }{2}.$$

Substituting Equation (A18) into Equation (A16) gives

(A19) $$\begin{array}{cc} k_{x}^{2}+k_{z}^{2} & =L_{s}C_{A}\omega^{2}-jR_{s}C_{A}\omega \\ & =-\left(R_{s}+j\omega L_{s}\right)j\omega C_{A}. \end{array}$$

Equation (A19) is the dispersion relation of the signal-transmission process in the AC-LGAD resistive layer. It shows that the propagation and attenuation characteristics are jointly determined by the sheet resistance, sheet inductance, coupling capacitance, and signal frequency.
